# The adult orthodontic patient over 40 years of age: association between periodontal bone loss, incisor irregularity, and increased orthodontic treatment need

**DOI:** 10.1007/s00784-021-03936-2

**Published:** 2021-04-22

**Authors:** Philipp Meyer-Marcotty, Daniela Klenke, Larissa Knocks, Petra Santander, Valentina Hrasky, Anja Quast

**Affiliations:** 1 Department of Orthodontics, University Medical Center Goettingen, Robert-Koch-Str. 40, 37075 Goettingen, Germany; 2 Department of Preventive Dentistry, Periodontology and Cariology, University Medical Center Goettingen, Robert-Koch-Str. 40, 37075 Goettingen, Germany

**Keywords:** Orthodontics, Periodontics, Adult, Treatment need, Bone loss

## Abstract

**Objectives:**

Adult orthodontic treatment, especially in patients over 40 years, is steadily increasing. One causal factor for the treatment need in this age group is periodontal breakdown. The aim of this study was to detect correlations between periodontal problems and orthodontic parameters in interdisciplinary patients.

**Methods:**

This observational, cross-sectional study included 118 patients over 40 years (51 men/67 women; mean age, 58.03 years) classified into three groups according to periodontal breakdown (group I, controls; group II, moderate periodontitis; group III, severe periodontitis). Clinical periodontal and orthodontic parameters as well as the index of orthodontic treatment need (IOTN) were assessed and compared between the groups.

**Results:**

A gradual deterioration of all periodontal and orthodontic parameters according to periodontal bone loss (lowest values in group I; highest values in group III) was observed. Especially groups I and III differed significantly regarding the overjet (*p* < 0.001) and the little indices of the maxilla (*p* < 0.001) and mandible (*p* < 0.010). The IOTN was highest in group III: 90% of the patients with severe periodontitis were classified to have moderate to very great treatment need.

**Conclusions:**

The higher the degree of periodontal breakdown was, the more severe were overjet, overbite, irregularity of the anterior teeth, and the orthodontic treatment need.

**Clinical relevance:**

Adult patients over 40 years represent a challenge for an orthodontic/periodontal treatment approach with high incidence of pathologic tooth migration, orthodontic treatment need, and periodontal breakdown. Therefore, this special patient collective requires a focus in clinical orthodontics and research.

## Introduction

Orthodontic therapy in adults is currently on the increase [1], even if it is not new. In 1723, Pierre Fauchard, one of the first systematic scientists in dentistry, published his observation that straightening teeth is more difficult in adults compared to children [2]. In the fifties of the past century, a thesis for board certification by the American Board of Orthodontics focused on adult therapy; it concluded that implementation of adult therapy is important and necessary to broaden the orthodontic spectrum [2]. Furthermore, an orthodontist named Victor Hugo Jackson already discussed successful treatment of adult patients from 40 to 50 years in a textbook from 1904 [2]. Today, these challenging patients (≥ 40 years of age) are no longer a rarity in the daily orthodontic practice and are more and more interested in an orthodontic correction of their accompanying malocclusion [3].

According to Melsen, in adult orthodontics, we must distinguish between “young adults” who should have been treated earlier and ”older adults” over 40 years of age with signs of aging and/or gradual deterioration [4]. These patients often present with a secondary malocclusion that has worsened or developed in adulthood. One causative factor could be a deterioration of the dentition and the periodontium [5]. Against this background, a close network between orthodontics and periodontics is becoming increasingly important for a successful treatment approach [6–9].

One linking element in diagnostics of both disciplines is pathologic tooth migration (PTM), which is defined as a change in tooth position that occurs when there is a disruption of forces that maintain teeth in a normal relationship. This definition suggests a multifactorial pathophysiology causing tooth migration. A characteristic clinical symptom is incisor flaring [10]. With a reported prevalence of 55.8% in periodontal patients, the migration, especially of anterior teeth, often represents the primary motivation for adults to consult an orthodontic practice [11].

The main factors known to influence tooth position are the tissues of the periodontium; occlusal factors; soft tissue pressures of the cheek, tongue, and/or lips; and a variety of oral habits. Proffit describes this as “equilibrium theory,” which means that all forces affecting the immediate surroundings of a tooth have to be balanced [12]. Keeping this in mind, the interaction between orthodontic malocclusion and periodontal bone level is of special interest for the orthodontic/periodontal scientific community. In recent literature, the need for quantification between malocclusion and periodontal bone loss in adult patients is emphasized [13].

Remarkably, there is a paucity of orthodontic literature on the potentially increasing treatment need of adults at an advanced age. The high prevalence of PTM in these patients leads to the hypothesis that there is a relationship among different degrees of periodontal bone loss, incisor irregularity, and orthodontic treatment need. Therefore, the aims of this study were:

- To detect periodontal and orthodontic parameters in interdisciplinary patients at an advanced age
- To analyze the impact of alveolar bone loss on incisor irregularity
- To quantify the adult orthodontic treatment need in relation to periodontal bone loss

## Patients and methods

This exploratory, observational, cross-sectional study was approved by the Institutional Ethics Committee of the Medical Faculty of the University of Goettingen (ethics number 3/1/17). The study was carried out according to the Declaration of Helsinki. All patients participated in the study on a voluntary basis after receiving comprehensive information about the aims and design of the study and signed an informed consent. This report complies with STROBE guidelines for observational studies [14].

### Patients

One hundred twenty-six adult patients were screened for participation in this study. All patients were recruited from the Section of Periodontology of the Department of Preventive Dentistry and referred to the Department of Orthodontics for data acquisition at the University Medical Center Goettingen. The data collection lasted from February 2017 to March 2018; the data were then analyzed by summer 2019 by one single investigator (L.K.). Of the 126 patients initially screened, 118 patients (51 men and 67 women, mean age: 58.03 years) were finally included in the study. The dropouts were due to lack of interest in orthodontics, missing teeth in the front, or an underlying severe malocclusion needing an orthognathic intervention. No additional controls were included after initial data acquisition to avoid potential bias.

The inclusion criteria were age ≥ 40 years, the presence of six anterior natural teeth in the upper and lower jaw, and no history of trauma. Exclusion criteria were an anterior open bite, history of cleft lip or palate, or a congenital syndrome.

The patients were classified into three groups according to their periodontal disease. Based on the CDC Periodontal Disease Surveillance Working Group, the categories were [15]:

- Group I—control group:
  Neither moderate nor severe periodontitis
- Group II—moderate periodontitis:
  ≥ 2 interproximal sites with clinical attachment loss (CAL) ≥ 4mm or pocket depth (PD) ≥ 5mm
- Group III – severe periodontitis:
  ≥ 2 interproximal sites with CAL ≥ 6mm and ≥ 1 interproximal site with PD ≥ 5mm

**Fig. 1:**
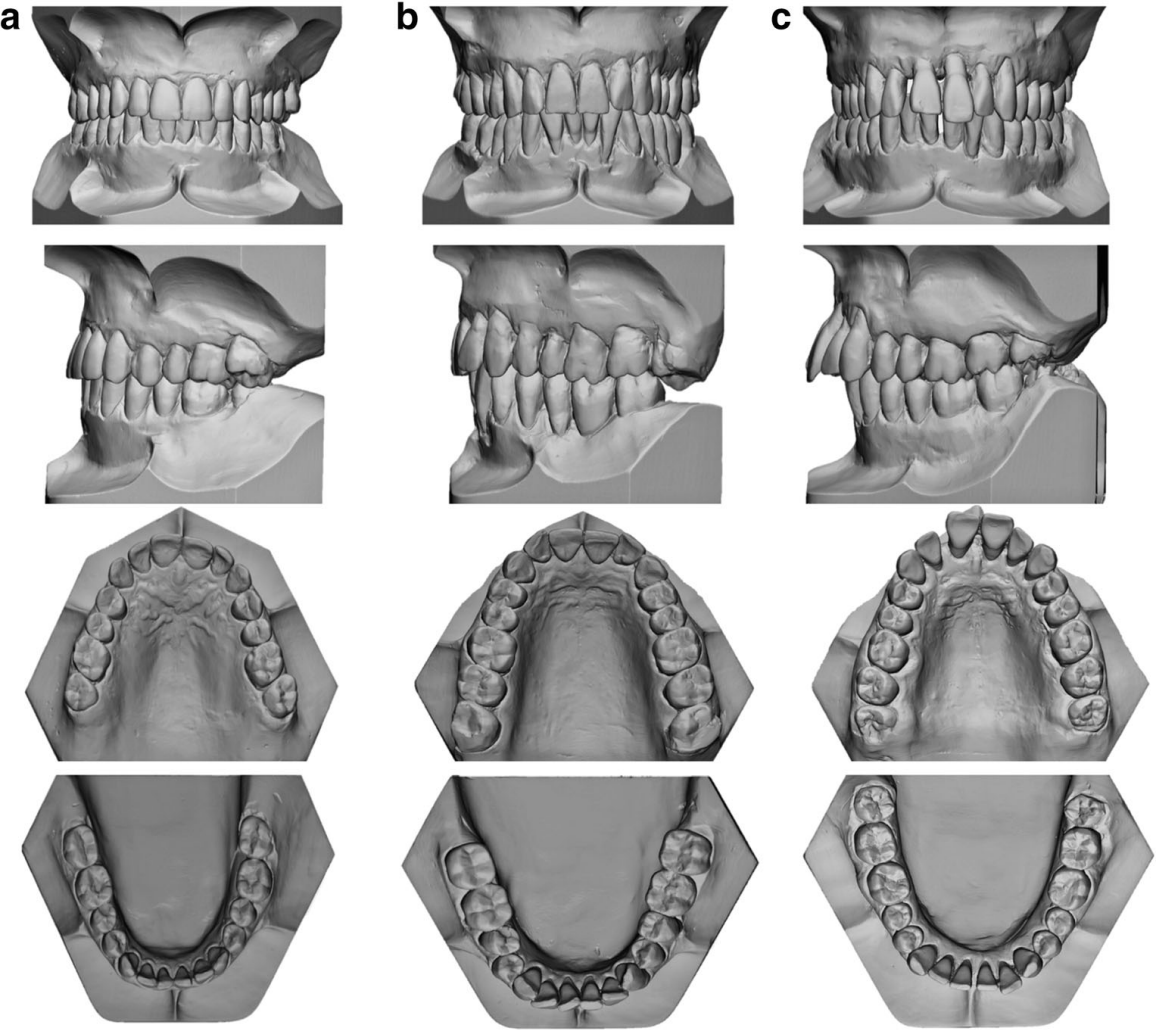
Three-dimensional digital dental cast of the three analyzed groups classified according to their periodontal bone loss. **a** Group I: control group—neither moderate nor severe periodontitis. **b** Group II: moderate periodontitis—≥ 2 interproximal sites with CAL ≥ 4mm or PD ≥ 5mm. **c** Group III: severe periodontitis—≥ 2 interproximal sites with CAL ≥ 6mm and ≥ 1interproximal site with PD ≥ 5mm. From a to c, the overjet, the severity of malocclusion, incisor irregularity, and orthodontic treatment need increase

In each group, periodontal and orthodontic measurements were performed on each tooth from canine to canine in the upper and lower jaw. Figure 1 displays exemplary digitized orthodontic casts for the three patient groups.

### Periodontal clinical parameters

The same person (L.K.) assessed the periodontal clinical parameters for each patient. As metric measurements, pocket depth (PD), clinical attachment loss (CAL), and gingival recession (GR) were analyzed using a periodontal probe. Each tooth of the upper and lower anterior sextant was examined in six areas (mesial, medial, and distal on labial and lingual/palatal tooth surface). In sum, this resulted in 72 measurements per patient per parameter.

### Orthodontic digital model parameters

To analyze PTM of the anterior teeth, dental casts of the upper and lower jaws were obtained for each patient. For a standardized analysis, the plaster casts were digitized in maximal intercuspidation with a stereophotogrammetric scanner (Zirkonzahn Scanner S300 Ortho (Zirkonzahn S.R.L., Gais, Italy)). The analysis was performed using the 3D software 3-matic Research 13.0 (Materialise N.V., Leuven, Belgium). The orthodontic digital model parameters were the overjet and overbite—assessed at the most severe side parallel and perpendicular to the occlusal plane respectively. To quantify the severity of malocclusion, the irregularity index of the mandible was defined by summing up the horizontal measurements of all (five) anatomic contact points between the incisors [16]. The same method was transferred to the maxillary anterior segment.

Additionally, the dental health component (DHC) of the index of orthodontic treatment need (IOTN) [17] was used to objectively quantify the severity of malocclusion. The IOTN-DHC grades the indications for treatment considering potential deleterious effects of malocclusion on functional and dental health. It was assessed by one author (L.K.) only, who was trained and experienced in the use of this ordinal scaled index.

### Statistics

Statistical analysis was performed using SPSS Statistics (v.25, IBM, New York, USA). For the orthodontic and periodontal parameters, the mean and 95% confidence intervals were calculated. To analyze statistical differences between the three groups (controls, patients with moderate periodontitis, and patients with severe periodontitis) a Kruskal-Wallis one-way analysis of variance was performed. Statistically significant differences were followed-up by Mann-Whitney *U* test to determine which groups contribute to this effect. All results were Bonferroni corrected. Group differences in the IOTN-DHC were analyzed by the Fisher’s exact test. The association between the Angle class and the degree of periodontal disease was tested by *χ*^2^ test. The level of significance was set at *p* < 0.05. To determine the error of the method, ten randomly selected 3D data sets were measured again by the same examiner after a 1-week interval, and the Spearman correlations coefficient was calculated. The maximum method error, i.e., the worst Spearman correlation coefficient, was 0.912 (*p* < 0.001) and was clinically irrelevant.

## Results

The demographic data for all patients are shown in Table 1. There was no statistically significant difference between the three groups according to their age (*p* = 0.071). The sex distribution in all three groups was well balanced. An orthodontic treatment during adolescence was documented in 33% of the patients. Angle classes were equally distributed between the groups (Cramer’s V: 0.123; *p* = 0.46) with the majority displaying class I.

**Table 1:** Descriptive statistics for all patients according to their group (group I, controls without moderate/severe periodontitis; group II, patients with moderate periodontitis; group III, patients with severe periodontitis)

Patients’ characteristics	Group I (*n*=39) Controls neither moderate nor severe periodontitis	Group II (*n*=39) Patients with moderate periodontitis	Group III (*n*=40) Patients with severe periodontitis	∑
Age (years)	57.36 (40; 78)	60.97 (40; 84)	55.83 (41; 72)	58.03 (40; 84)
Sex				
Men	31%	46%	52.5%	43%
Women	69%	54%	47.5%	57%
History of orthodontic treatment in adolescence	31%	21%	48%	33%
Angle classification				
Class I	72%	69%	62.5%	68%
Class II	25.5%	26%	37.5%	29.5%
Class III	2.5%	5%	0%	2.5%

For age, the mean (minimum; maximum) were reported; for sex, history of orthodontic treatment and angle classification the frequency was calculated; ∑ = total

n.s. = not significant

The results of the descriptive analysis of the periodontal and orthodontic parameters for all patients are demonstrated in Table 2. Statistically significant differences were found for all periodontal parameters among the three different groups. The values of the periodontal parameters increased stepwise with the lowest values for the controls (group I) and the highest values in group III (patients with severe periodontitis). Only the average of recession in the mandible was slightly higher in group II with a mean of 0.55 mm versus 0.38 mm in group III. A similar pattern with a gradual increase of the values was found for all orthodontic parameters—again with the lowest values for the controls (group I) and the highest values in group III (patients with severe periodontitis). A significant intergroup difference could be demonstrated for the overjet, Little Index of the mandible, and the maxilla. No significant effect was detected for the overbite.

**Table 2:** Descriptive statistics of the periodontal clinical and the orthodontic digital parameters according to their group (group I, controls without moderate/severe periodontitis; group II, patients with moderate periodontitis; group III, patients with severe periodontitis) and corresponding results of the Kruskal-Wallis one-way analysis of variance for the overall comparison between all groups followed up by Mann-Whitney *U* test for intergroup analysis

Periodontal clinical parameters		Group I (*n*=39) Controls neither moderate nor severe periodontitis		Group II (*n*=39) Patients with moderate periodontitis		Group III (*n*=40) Patients with severe periodontitis		Overall comparison (group I vs. II vs. III)	Intergroup comparison		
									Group I vs. II	Group I vs. III	Group II vs III
	Unit	Mean	95 % CI	Mean	95 % CI	Mean	95 % CI	*p*	*p*	*p*	*p*
Pocket depth max^+^	mm	1.68	[1.56–1.79]	1.91	[1.77–2.05]	2.96	[2.69–3.22]	< 0.001***	0.012*	< 0.001***	< 0.001***
Pocket depth mand^++^	mm	1.42	[1.33–1.51]	1.58	[1.45–1.71]	2.56	[2.29–2.83]	< 0.001***	0.076 n.s.	< 0.001***	< 0.001***
Recession max^+^	mm	0.03	[0.01–0.05]	0.30	[0.18–0.43]	0.37	[0.27–0.46]	< 0.001***	< 0.001***	< 0.001***	0.105
Recession mand^++^	mm	0.07	[0.04–0.10]	0.55	[0.36–0.73]	0.38	[0.29–0.48]	< 0.001***	< 0.001***	< 0.001***	0.533
CAL max^+^	mm	1.71	[1.59–1.82]	2.21	[2.08–2.34]	3.32	[3.02–3.61]	< 0.001***	< 0.001***	< 0.001***	< 0.001***
CAL mand^++^	mm	1.49	[1.40–1.58]	2.13	[1.96–2.29]	2.94	[2.65–3.24]	< 0.001***	< 0.001***	< 0.001***	< 0.001***
Orthodontic digital parameters											
Overjet	mm	2.60	[2.10–3.08]	3.37	[2.73–4.02]	4.38	[3.58–5.17]	< 0.001***	0.026*	< 0.001***	0.051 n.s.
Overbite	mm	2.95	[2.33–3.56]	3.10	[2.68–3.52]	3.45	[2.81–4.09]	0.475 n.s.	-	-	-
Little Index max^+^	mm	1.76	[1.22–2.30]	2.56	[1.77–3.36]	4.06	[2.96–5.16]	0.001**	0.511 n.s.	0.001**	0.062 n.s.
Little Index mand^++^	mm	2.71	[1.97–3.44]	3.78	[2.85–4.72]	4.33	[3.46–5.19]	0.012*	0.187 n.s.	0.010**	0.875 n.s.

The mean and 95% confidence Interval (CI) were reported; significance level was set at *p* < 0.05, adjusted by Bonferroni correction

^+^maxilla

^++^mandibula; *CAL* clinical attachment level

Kruskal-Wallis and Mann-Whitney-U test: *n.s*. not significant

*p < 0.05

**p < 0.01

***p < 0.001

The intergroup analysis detected significant differences for each group comparison for almost all periodontal parameters. Only the parameter “pocket depth in the mandible” between the control group and group II and the parameters “recession of the maxilla/mandible” between group II and group III showed no significance. The gradually increasing pattern leading to the greatest pathological expression in patients with severe periodontitis was confirmed. The analysis for the orthodontic parameters showed significant differences for the overjet in each group—comparison for group I vs. group II and for group I vs. group III. The lowest values in the control group and the highest values in group III once again confirmed the increasing pathological pattern. In contrast, the Little Indices of the maxilla and mandible were not significantly different between the control group and group II; but when comparing the control group with group III, significant higher values were found in patients with severe periodontitis. The analysis of group II vs. group III showed no significant difference for the orthodontic parameters.

Table 3 shows the results of the descriptive analysis and statistical differences for the IOTN. Group III revealed the highest value of the index. A significant difference for each group comparison was detected between the control group and group III (patients with severe periodontitis). The prevalence of the different IOTN classifications is listed in Table 4. Ninety percent of the patients with severe periodontitis were classified as grade 3 or higher, resulting in moderate to very great treatment need. In contrast, a moderate to very great treatment need was demonstrated in 74.4% of all patients in group II and only 59.1% in group I.

**Table 3:** Descriptive statistics for the Index of Orthodontic Treatment Need–Dental Health Category (IOTN-DHC) of all patients according to their groups (group I, controls without moderate/severe periodontitis; group II, patients with moderate periodontitis; group III, patients with severe periodontitis) and the results of the overall and intergroup comparison by Fisher’s exact test

Orthodontic digital parameter	Group I (*n*=39) Controls neither moderate nor severe periodontitis		Group II (*n*=39)Patients with moderate periodontitis		Group III (*n*=40)Patients with severe periodontitis		Overall comparison (group I vs. II vs. III)	Intergroup comparison		
								Group I vs II	Group I vs. III	Group II vs. III
	Mean	95% CI	Mean	95% CI	Mean	95 % CI	*p*	*p*	*p*	*p*
IOTN-DHC	2.85	[2.58–3.11]	3.18	[2.91–3.45]	3.45	[3.21–3.69]	0.022*	0.205	0.005**	0.165

The mean and 95% confidence interval (CI) were reported; significance level was set at *p* < 0.05, adjusted by Bonferroni correction

Fisher’s exact test: *n.s.* not significant

*p < .05

**p < .01

***p < .001

**Table 4:** Prevalence of the index of orthodontic treatment need (IOTN) classifications for the control group (group I), for patients with moderate periodontitis (group II), and for patients with severe periodontitis (group III)

Classification IOTN-value	Clinical implication	Prevalence in % group I	Prevalence in % group II	Prevalence in % group III
1	None	0	0	0
2	–	41	25.6	10
	Little			
3	Moderate	33.3	30.8	40
4	–	25.6	43.6	45
5	Very great	0	0	5

## Discussion

In line with the diversification of modern orthodontics, adult therapy is growing rapidly. Not only young adults in their twenties, but patients over 40 years of age are consulting orthodontists more frequently these days. The transition from children and adolescents as the traditional patient cohort in the daily orthodontic clinic to challenging adult patients is already in full swing. In view of this, two principles come more into focus for the orthodontist: (a) the periodontal and orthodontic interrelationship [18] and (b) the adult orthodontic treatment need [3].

In this clinical study, 118 orthodontic patients over 40 years of age were enrolled. Three groups of patients were assigned according to their clinical degree of bone loss, and their periodontal and orthodontic parameters were analyzed.

### Periodontal parameters

Using the CAL as the main periodontal parameter, the control group, the patients with moderate periodontitis, and the patients with severe periodontitis could be clearly separated from each other. A significant difference was shown in both the maxilla and in the mandible. This is in accordance with the working group of the Centers for Disease Control and Prevention and the American Academy of Periodontology (CDC/AAP) in 2003, where thresholds were defined for severe periodontitis with a CAL ≥ 6 mm and for moderate periodontitis with a CAL ≥4 mm [15, 19]. They defined the measurement of the CAL as the gold standard for diagnostics in periodontics. Furthermore, CAL revealed to be a more accurate measure of disease history and disease progression than PD alone. This is underpinned by the results of this study: a highly significant difference in PD in the mandible and maxilla was shown in patients with severe periodontitis vs. patients with moderate periodontitis and between the control group and patients with severe periodontitis. However, no significant difference was detectable between the control group and patients with moderate periodontitis in the mandible, and the difference in the maxilla was only marginal. This less indicative group classification by PD could be explained by the age of the patients enrolled in this study: Because the focus fell on patients over 40 years of age, no direct correlation between PD and CAL compared to younger adults could be analyzed. It has been shown that in (post-) middle age patients, gingival recessions increase more rapidly than PD [15]; therefore, CAL and PD no longer correlate in adults over the age of 40. In this respect, the parameters selected for the study were confirmed.

### Orthodontic parameters

The parameters overjet and overbite increased progressively in all three groups. Regarding the periodontal breakdown, the overjet was 30% higher in patients with moderate periodontitis and 69% higher in patients with severe periodontitis compared to the controls. The overbite was 5% and 17% higher, respectively. In summary, the higher the degree of periodontal breakdown, the more severe the overjet and overbite are in patients over 40 years of age. These results, which are typical findings in the daily clinical practice, were now quantified by our study in adult orthodontic patients. However, it has to be kept in mind that the overjet and overbite are also strongly associated with the Angle classes. A lack of incisor contact may favor dental elongation, especially in combination with periodontal breakdown. Therefore, we aimed for equally distributed Angle classification among the groups.

The herein observed pathophysiology is explained by the fact that the degeneration of the periodontium and the alveolar boundary results in PTM [20]. The clinical consequence is an initial PTM, which can be detected as “flaring out” of the upper frontal teeth in patients with severe periodontitis [21, 22]. This is substantiated by the findings in our study, in which a primary increase of “flaring out” automatically resulted in a higher overjet in patients with moderate to severe periodontitis. Thereby, an increase in periodontal disease seemed to be associated with deterioration of overjet but not overbite. These results are in accordance with recent published literature [23].

In addition to periodontal breakdown, another factor contributing to “flaring out” of the upper teeth could be the surrounding soft tissues (e.g., lower lip) [12]. Even though the surrounding forces by the soft tissues are very light, it has been documented that forces as light as 1.0 g produced by the facial tissue are sufficient to initiate displacement of upper or lower incisors [24]. Therefore, the lower lip has to be regarded as another progressive factor for developing more severe malocclusions: when the increased overjet is associated with lower lip dysfunction and periodontal bone loss, the upper incisors protrude more and more due to the lip pressure. Thus, a vicious circle (periodontal breakdown, flaring out, lip dysfunction) is developing in orthodontic patients with moderate/severe periodontitis.

Another orthodontic parameter which could be affected by periodontal bone loss is the irregularity of the anterior teeth—expressed by the Little Index. In our study, the Little Index increased in the maxilla and mandible stepwise from group I to group III, resulting in a significant difference between the controls and patients with severe periodontal bone loss. These findings are contrary to previous literature where no association was found between periodontal bone loss and crowding [25]. This could be attributed to the fact that in the present study, the age range of the included patients was more focused on ≥ 40 years of age, whereas the previous study included patients from 21 up to 55 years of age.

When comparing the Little Index between the maxilla and mandible, the mandible revealed more irregularity of the frontal teeth in each group. This is confirmed by recent literature showing irregularity in the mandibular incisor area to be associated with a distinct localized periodontal breakdown [26]. The reason could be the characteristic morphology with thin bone or previously existing fenestration/dehiscence and root proximity, resulting in a higher sensitivity to PTM.

However, the differences in the Little Index between both jaws waned with increasing bone loss. Patients with severe periodontitis had only a minimal difference between maxillary and mandibular irregularity of the frontal teeth. This effect may be explained by the different bone architecture in the maxilla with more cancellous bone, even in the anterior alveolar region [27, 28]. This anterior alveolar region seems more sensitive to PTM when bone loss is increasing. Thus, by exceeding a certain threshold of bone loss, a similar pathological mesial migration of the teeth is observed in the mandible and maxillary arch.

### Index of orthodontic treatment need

Up to now, there is no data describing the orthodontic treatment need of adults in a focused patient group over 40 years of age with differing degrees of bone loss. Only one study has investigated the orthodontic treatment need using the esthetic component (AC) of the IOTN in a similar patient cohort (mean age 56.8 years; moderate to severe periodontitis) [3]. In this former study, one-third of the patients showed a moderate to definite treatment need; but this study focused only on dental attractiveness, i.e., the esthetic component. In contrast, the present study analyzed the Dental Health Component (DHC) of the IOTN taking potential deleterious effects of malocclusion on the health and functioning of the dentition into account.

The IOTN-DHC revealed a significantly higher treatment need in patients with severe periodontitis compared to patients with moderate/no periodontal bone loss. Again, this may be associated with the statistically higher degree of PTM because of severe periodontal bone loss [29]. Moreover, it is known that the risk of PTM rises by a factor of more than 2.5 if there is an increase in bone loss. This uncontrolled tooth migration results in a higher IOTN classification, so that 90% of the patients with severe bone loss were classified as grade 3 or higher, which means moderate to very great treatment need. Keeping in mind that the IOTN-DHC has a reproducibility of 93% [17], these data indicate the necessity for a reorientation in adult orthodontic treatment, focusing on patients with advanced age.

## Conclusions

Adult patients with an advanced age represent a challenge for interdisciplinary orthodontic/periodontal treatment approaches. The findings of our study with patients over 40 years of age showed that the higher the degree of periodontal breakdown is,

- The more severe is the overjet and overbite
- The more severe is the irregularity of the anterior teeth in the maxilla and the mandible
- The higher is the reported treatment need compared to patients with no periodontal bone loss.

Therefore, this special patient cohort will require a particular focus in clinical orthodontics and research in the near future.

## Abbreviations

PTM: pathologic tooth migration
PD: pocket depth
CAL: clinical attachment loss
GR: gingival recession
DHC: dental health component
IOTN: index of orthodontic treatment need
CDC: Centers for Disease Control and Prevention
AAP: American Academy of Periodontology
AC: esthetic component
